# Alliin Regulates Intracellular Cholesterol Metabolism Through the PCSK9/LDLR Axis in Ox‐LDL‐Induced HepG2 Cells: A Proteomics Study

**DOI:** 10.1002/fsn3.72148

**Published:** 2026-07-24

**Authors:** Yuanyuan Tang, Xiaoshi Liu

**Affiliations:** ^1^ Department of Pharmacy Hospital of Chengdu University of Traditional Chinese Medicine Chengdu China; ^2^ Department of Gynecologic Oncology Sichuan Clinical Research Center for Cancer, Sichuan Cancer Hospital & Institute, Sichuan Cancer Center, University of Electronic Science and Technology of China Chengdu Sichuan China; ^3^ School of Medicine University of Electronic Science and Technology of China Chengdu Sichuan China

**Keywords:** alliin, HepG2 cells, lipid accumulation, PCSK9/LDLR pathway, proteomics

## Abstract

This study focuses on alliin, a bioactive compound derived from garlic, and investigates its underlying mechanisms through a proteomics‐based approach. In HepG2 cells, alliin significantly reduced intracellular total cholesterol and triglyceride levels. Tandem mass tag‐based quantitative proteomic analysis identified 67 differentially expressed proteins (53 upregulated, 14 downregulated). Bioinformatics enrichment revealed these proteins were primarily involved in cholesterol metabolism, the PPAR signaling pathway, and the PI3K/Akt pathway. Notably, alliin significantly upregulated low‐density lipoprotein receptor (LDLR) expression while downregulating proprotein convertase subtilisin/kexin type 9 (PCSK9). Western blot validation confirmed that alliin modulates the PCSK9/LDLR pathway. In conclusion, alliin influences intracellular cholesterol metabolism in HepG2 cells, potentially through regulation of the PCSK9/LDLR pathway. These findings provide mechanistic insights at the cellular level and suggest that alliin may serve as a candidate compound for further investigation in the context of lipid metabolism–related disorders.

## Graphical abstract

This study demonstrates that alliin reduces intracellular lipid accumulation and alters protein expression in ox‐LDL‐induced HepG2 cells. These effects are linked to cholesterol metabolism and the PPAR and PI3K/Akt signaling pathways. Mechanistically, alliin regulates the PCSK9/LDLR axis, indicating its role in maintaining intracellular cholesterol homeostasis.

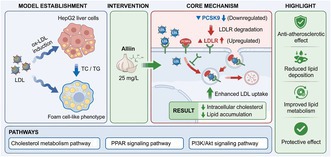

## 1 Introduction

Atherosclerotic (AS) lesions constitute the primary pathological basis underlying the high incidence and mortality of cardio‐cerebrovascular diseases (CCVD) (Goldsborough III et al. 2022; Hisamatsu and Kinuta 2023). AS represents a major threat to human health and survival. Its initiation and progression involve a complex pathophysiological process characterized by vascular endothelial dysfunction, increased oxidative stress, abnormal lipid accumulation in the subendothelial layer, and chronic inflammatory responses mediated by cytokines such as tumor necrosis factor‐α (TNF‐α) and interleukin‐1β (IL‐1β). In addition, monocytes/macrophages engulf modified lipoproteins and subsequently transform into foam cells, which further drive lesion development (Greenland and Lloyd‐Jones 2022; Yu, Alimujiang, et al. 2021; Weitgasser et al. 2018). The progression of AS is closely associated with plasma levels of total cholesterol (TC) and triglycerides (TG). Elevated plasma lipid levels promote excessive lipid infiltration into the arterial wall, leading to local lipid deposition, proliferation of vascular smooth muscle cells, and foam cell formation, thereby establishing a critical pathological foundation for the initiation and progression of AS (Vance 2022; Sobati et al. 2020; Li et al. 2021; Bäck et al. 2019).

In traditional Chinese medicine, garlic has long been utilized for the prevention and treatment of atherosclerosis‐related diseases (Valls et al. 2022). In recent years, modern pharmacological studies have provided molecular‐level evidence supporting its traditional functions of “resolving phlegm and removing blood stasis.” Garlic has been shown to contain more than 10 natural organosulfur compounds, the majority of which are derived from L‐cysteine sulfoxide and γ‐glutamyl‐L‐cysteine peptides (Liu et al. 2022).

Fresh garlic is particularly rich in alliin (S‐allyl‐L‐cysteine sulfoxide), an important precursor of these bioactive components. In intact garlic cloves, alliin remains stable within the cytoplasm. However, when the cloves are cut or crushed, alliin is rapidly converted by alliinase into allicin, a compound responsible for garlic's characteristic pungent odor and a key contributor to its biological activity (Nawaka et al. 2022).

Although the lipid‐lowering effects of garlic have been widely recognized, studies specifically investigating alliin in the context of atherosclerosis—particularly those involving HepG2 cells—remain relatively limited, based on literature retrieved from PubMed, Google Scholar, and Web of Science using the keywords “alliin–HepG2 cells–atherosclerosis.” For instance, Siegers et al. (Rozanova et al. 2021) reported that garlic powder containing approximately 10% alliin significantly inhibited the proliferation of both HepG2 cells and human rectal cancer cells. Similarly, Lu et al. (2017) demonstrated that alliin markedly ameliorated lipid metabolism disorders induced by 1,3‐dichloro‐2‐propanol (1,3‐DCP) in HepG2 cells. In our earlier publication, we investigated the protective effects of alliin on ox‐LDL‐induced injury in HUVECs, focusing on endothelial dysfunction (Tang and Lv 2025). However, the role of alliin in hepatocytes, which are central to lipid metabolism and lipoprotein regulation, remains unclear (Shi et al. 2017; Yamauchi et al. 2026; Yang et al. 2024). Compared with our previous study, our current study takes alliin as the research object; systematically explores the effects of alliin on lipid accumulation and proteomic alterations in ox‐LDL‐injured HepG2 cells, with a focus on pathways potentially involved in lipid metabolism regulation; and aims to provide new experimental evidence and theoretical support for the prevention of AS through the application of traditional Chinese medicine.

## 2 Methods

### 2.1 Drugs and Reagents

Alliin (lot no. 202044) was obtained from Beijing Solarbio Technology Co. Ltd. Dulbecco's Modified Eagle's Medium (DMEM; lot no. 2018035) and fetal bovine serum (FBS; lot no. 202018RP) were obtained from Gibco. The Cell Counting Kit‐8 (CCK‐8; lot no. AR1160) and polyvinylidene difluoride (PVDF) membranes were supplied by Boster Biological Technology Co. Ltd. The TC assay kit (lot no. A111‐1‐1, 25 mL) and TG assay kit (lot no. A110‐1‐1, 25 mL) were obtained from Nanjing Jiancheng Bioengineering Institute. Phosphate‐buffered saline (PBS, 1×), 0.5% trypsin solution, and penicillin–streptomycin were purchased from HyClone. Six‐ and 96‐well culture plates were obtained from Biosharp Biotechnology Co. Ltd. Primary antibodies against LDLR (ab52818), PCSK9 (ab272907), and β‐actin (ab8226), as well as secondary anti‐rabbit (ab6702) and anti‐mouse (ab150113) antibodies, were purchased from Abcam (Cambridge, MA, USA). The bicinchoninic acid (BCA) protein assay kit (lot no. P0009) was obtained from Beyotime Biotechnology.

### 2.2 Maintenance and Culture of Cells

HepG2 cells were obtained from the Cell Bank of the Chinese Academy of Sciences (Shanghai, China) and used at passages 3–10 for all experiments. The cell line was authenticated by short tandem repeat profiling and routinely tested to confirm the absence of mycoplasma contamination. HepG2 cells were maintained in DMEM supplemented with 15% FBS and 1% penicillin–streptomycin and incubated under standard conditions (37°C, 5% CO_2_). For subsequent experiments, cells were seeded into 6‐well plates and allowed to grow until approximately 70% confluence before further treatment.

### 2.3 Assessment of the Effects of Alliin on HepG2 Cell Viability

Cell viability was assessed using the CCK‐8 assay. HepG2 cells were seeded into 96‐well plates at a density of 6 × 10^3^ cells per well and incubated overnight. Cells were then exposed to increasing concentrations of alliin (12.5, 25, 50, and 100 mg/L) for 24 h, while untreated cells served as controls. After treatment, 10 μL CCK‐8 solution was added and incubated for 30 min in the dark. Absorbance was measured at 450 nm using a microplate reader (Bio‐Rad Laboratories, Hercules, CA, USA). Relative cell viability was calculated relative to the control group, and experiments were conducted in quintuplicate.

### 2.4 Determination of Lipid Accumulation and TC/TG Levels in HepG2 Cells

The experiment consisted of two groups: a model group treated with 80 μg/mL ox‐LDL alone and an experimental group co‐treated with 80 μg/mL ox‐LDL and 25 mg/L alliin. Each condition was performed in triplicate. After 24 h of incubation, the TC and TG levels in the two groups were determined using commercial assay kits according to the manufacturer's protocols and normalized to protein concentration. For visualization of intracellular lipid droplets, HepG2 cells were divided into three groups: a control group treated with 80 μg/mL ox‐LDL alone, and two experimental groups co‐treated with 80 μg/mL ox‐LDL and alliin at concentrations of 25 and 50 mg/L. After treatment, the HepG2 cells were fixed with paraformaldehyde and stained with Nile Red. After washing to remove excess dye, representative fluorescence images were captured using a fluorescence microscope (IX73, Olympus Corporation, Tokyo, Japan) under identical exposure settings for all groups.

### 2.5 Bioinformatics Analysis

HepG2 cells were divided into three groups: a control group, a model group treated with 80 μg/mL ox‐LDL, and a treatment group exposed to 80 μg/mL ox‐LDL in combination with 25 mg/L alliin. Protein samples were extracted from these three groups and then subjected to enzymatic digestion. After that, the resulting peptides were analyzed by LC–MS/MS. Protein identification and quantification were performed using MaxQuant software (version 1.6.17.0) based on unique peptides. Differentially expressed proteins between the alliin‐treated and model groups were identified using the thresholds of *p* < 0.05, and log_2_ fold change > 0.5 for upregulated proteins, and *p* < 0.05 and log_2_ fold change < −0.5 for downregulated proteins. Functional annotation was conducted using Gene Ontology (GO) and Kyoto Encyclopedia of Genes and Genomes (KEGG) databases. The protein–protein interaction network was constructed by the STRING database and visualized using R‐based tools (version 4.2.0).

### 2.6 Western Blot Analysis of LDLR and PCSK9 Expression in HepG2 Cells

HepG2 cells were assigned to two groups: a model group and an alliin‐treated group (25 mg/L). Total protein was extracted by lysing cells in 300 μL of RIPA buffer on ice, followed by centrifugation at 12,000 × g for 10 min at 4°C. The supernatant was collected. Protein concentrations were measured using a BCA assay kit in accordance with the manufacturer's instructions. Equal amounts of protein (80 μg) were denatured at 85°C for 10 min, separated by 8% SDS–PAGE for 1 h, and subsequently transferred onto PVDF membranes. The membranes were blocked with 5% non‐fat milk in TBST for 1 h at room temperature before being incubated overnight at 4°C with primary antibodies against LDLR, PCSK9, and β‐actin (all diluted 1:1000). Following washing, the membranes were incubated with suitable HRP‐conjugated secondary antibodies (anti‐rabbit or anti‐mouse, 1:5000 dilution) for 2 h at room temperature. Protein bands were observed using an enhanced chemiluminescence system (ChemiDoc XRS+, Bio‐Rad Laboratories, Hercules, CA, USA), and protein expression levels were normalized to β‐actin.

### 2.7 Statistical Analysis

Data are expressed as mean ± standard deviation (SD) and analyzed with GraphPad Prism 10.1.2. One‐way analysis of variance (ANOVA) was first conducted for intergroup comparisons. Dunnett's post hoc test was applied when all experimental groups were compared against a single control group, whereas Tukey's HSD post hoc test was used for all pairwise comparisons among multiple experimental groups. Statistical significance was set at *p* < 0.05, with *p* < 0.01 considered highly significant.

## 3 Results

### 3.1 Effects of Alliin on Proliferation and Lipid Accumulation in HepG2 Cells

**FIGURE 1:**
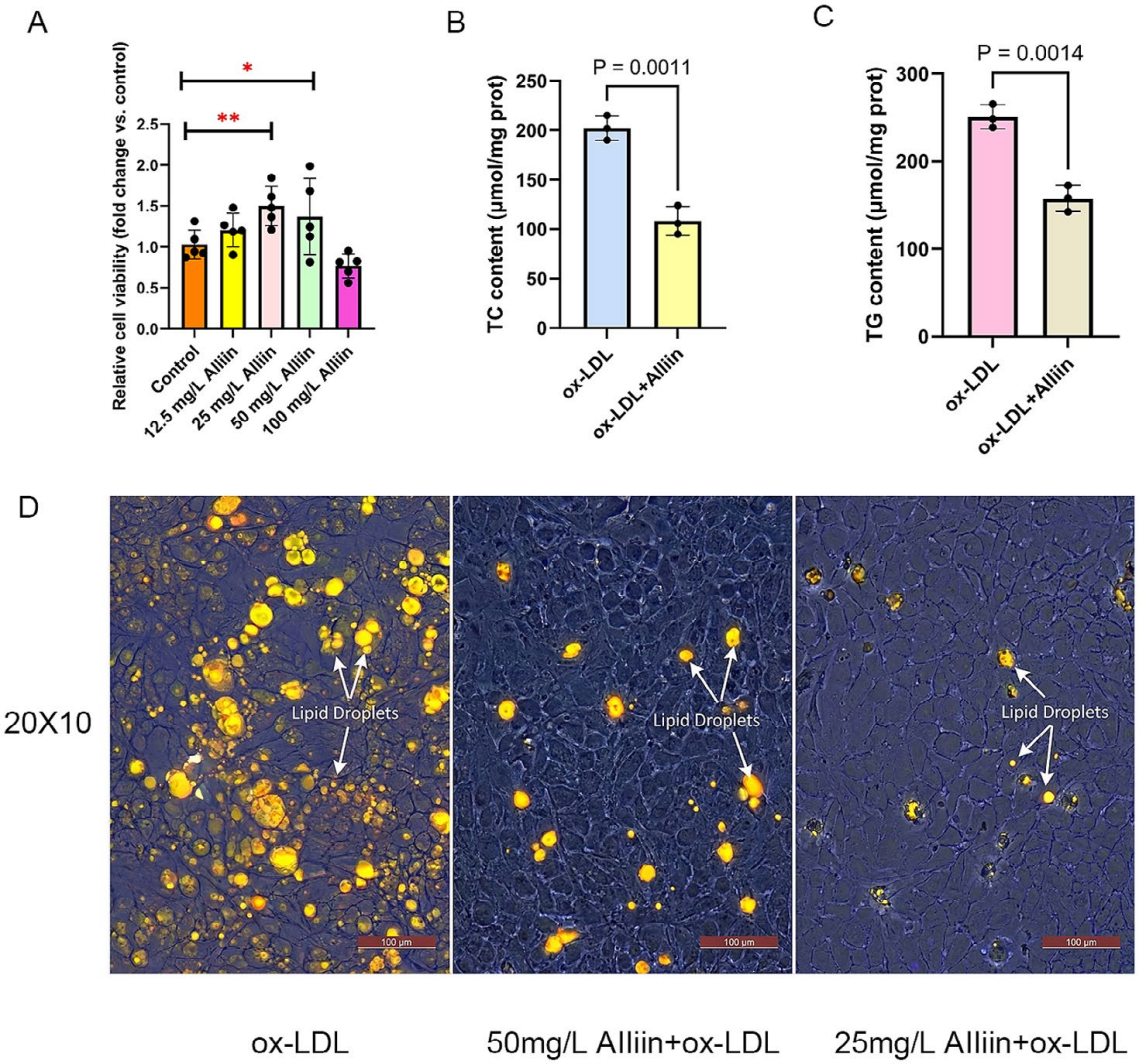
Effects of alliin on HepG2 cells. (A) The effect of alliin (12.5–100 mg/L) on the proliferation of HepG2 cells after 24 h was evaluated using the CCK‐8 assay. Relative cell viability was expressed as fold change normalized to the control group. Cells cultured in DMEM alone served as the control group. Data are presented as mean ± S.D. (*n* = 5). *p < 0.05, **p < 0.01 vs. the control group. (B) TC levels in HepG2 cells in the ox‐LDL‐induced injury group and alliin‐treated groups. The ox‐LDL–treated group served as the control. Data are presented as mean ± S.D. (*n* = 3). **p < 0.01 vs. ox‐LDL group. (C) TG levels in HepG2 cells in the ox‐LDL‐induced injury group and alliin‐treated groups. The ox‐LDL–treated group served as the control. Data are presented as mean ± S.D. (*n* = 3). **p < 0.01 vs. ox‐LDL group. (D) Lipid accumulation in HepG2 cells as determined by Nile Red staining.

The effect of alliin (12.5–100 mg/L) on the proliferative activity of HepG2 cells at 24 h was evaluated using the CCK‐8 assay. As shown in Figure 1A, treatment with alliin at concentrations of 25–50 mg/L for 24 h significantly enhanced relative cell viability compared with the control group (*p* < 0.05), with the most pronounced effect observed at 25 mg/L (*p* < 0.01).

After 24 h of treatment with 25 mg/L alliin, intracellular TC and TG levels were measured. As illustrated in Figure 1B,C, alliin treatment significantly reduced TC and TG contents in HepG2 cells compared with the ox‐LDL‐treated group (*p* < 0.01), which served as the reference group for these assays.

Furthermore, Nile Red staining was performed to visualize intracellular lipid accumulation. As shown in Figure 1D, compared with the ox‐LDL–injured HepG2 cell group, treatment with alliin (25 and 50 mg/L) markedly reduced lipid droplet deposition in HepG2 cells, whereas treatment with alliin (25 mg/L) notably reduced lipid droplet accumulation, indicating a significant attenuation of lipid accumulation.

Additionally, different control conditions were used across panels to match the experimental aims. Panel A used a DMEM‐only control to assess basal cell viability. In contrast, panels B–D used the ox‐LDL–treated group as the reference to evaluate the protective effects of alliin on lipid metabolism.

### 3.2 Quantitative Analysis of Differentially Expressed Proteins and Subcellular Localization

**FIGURE 2:**
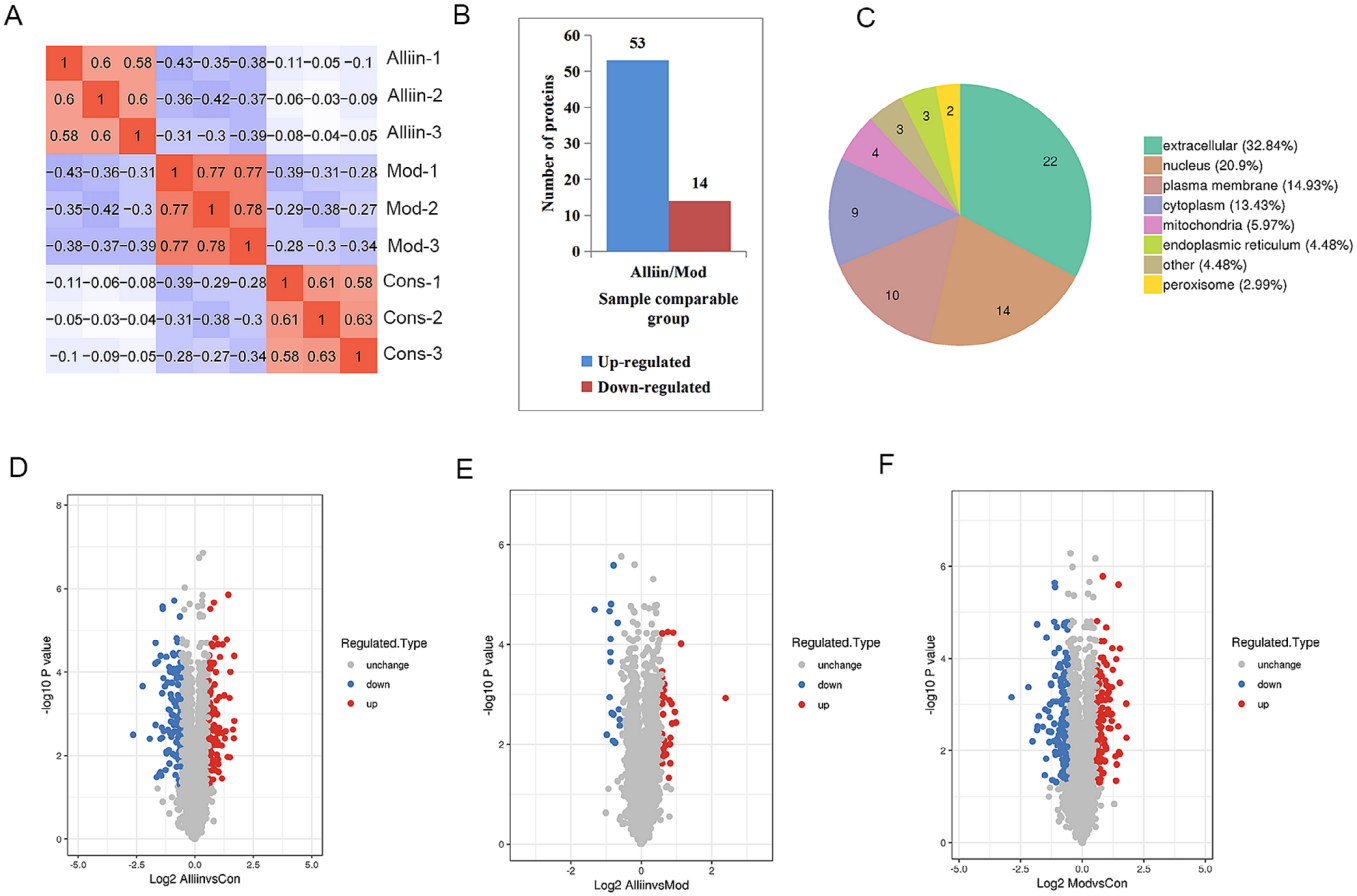
Proteomic analysis of differentially expressed proteins. (A) Pearson correlation analysis. (B) Differential expression analysis between the Alliin‐treated group and the model group induced by 80 μg/mL ox‐LDL. (C) Subcellular localization analysis of differentially expressed proteins. (D–F) Volcano plots of differentially expressed proteins.

Figure 2A from the Pearson correlation analysis showed strong reproducibility among the samples, including the Alliin‐treated groups (Alliin 1–3), control groups (Con 1–3), and model groups (Mod 1–3). The correlation coefficients within each group were close to 1, indicating high consistency and reliability of protein quantification across biological replicates. Differential expression analysis between the Alliin‐treated group and the model group induced by 80 μg/mL ox‐LDL identified 53 upregulated proteins and 14 downregulated proteins.

Table 1 summarizes 22 representative differentially expressed proteins, including their gene symbols, corresponding protein names, Log2 fold changes (Log2Ratio), regulation trends, and associated pathways. Notably, proteins related to cholesterol biosynthesis and extracellular matrix organization were significantly upregulated, suggesting that metabolic activation and structural remodeling are key biological responses. The complete MS‐based protein identification information is provided in Table S1.

**TABLE 1:** Protein expression profile table.

ID	Gene symbol	Protein name	Log2Ratio	Regulation	Pathway association
P99999	CYCS	Cytochrome c	0.653▲	Up*	Apoptosis
Q9UBM7	DHCR7	7‐dehydrocholesterol reductase	0.755▲	Up*	Cholesterol synthesis
O43493	TGOLN2	Trans‐Golgi network protein 2	−0.683▼	Down*	Vesicle transport
P01130	LDLR	Low‐density lipoprotein receptor	0.586▲	Up*	Lipid metabolism
P00488	F13A1	Coagulation factor XIII A chain	0.697▲	Up*	Blood coagulation
Q15800	MSMO1	Methylsterol monooxygenase 1	1.118▲	Up*	Cholesterol synthesis
P00738	HP	Haptoglobin	0.899▲	Up*	Acute‐phase response
O43451	MGAM	Maltase‐glucoamylase	0.641▲	Up*	Carbohydrate digestion
P02751	FN1	Fibronectin	0.647▲	Up*	Extracellular matrix
Q14534	SQLE	Squalene monooxygenase	1.031▲	Up*	Cholesterol synthesis
P04196	HRG	Histidine‐rich glycoprotein	0.989▲	Up*	Angiogenesis
P02656	APOC3	Apolipoprotein C‐III	0.996▲	Up*	Lipid transport
P37268	FDFT1	Squalene synthase	0.722▲	Up*	Cholesterol synthesis
Q13907	IDI1	Isopentenyl‐diphosphate isomerase	0.651▲	Up*	Cholesterol synthesis
P07996	THBS1	Thrombospondin‐1	0.628▲	Up*	Extracellular matrix
Q16610	ECM1	Extracellular matrix protein 1	0.698▲	Up*	Extracellular matrix
Q01581	HMGCS1	HMG‐CoA synthase	0.756▲	Up*	Cholesterol synthesis
Q96JB1	DNAH8	Dynein heavy chain 8	1.198▲	Up*	Cilium movement
P02795	MT2A	Metallothionein‐2	−0.626▼	Down*	Metal ion binding
P05106	ITGB3	Integrin beta‐3	−0.730▼	Down*	Cell adhesion
P02790	HPX	Hemopexin	0.757▲	Up*	Heme transport
Q12899	TRIM26	Tripartite motif‐containing 26	0.644▲	Up*	Immune response

*Note:* Key annotations: ▲, upregulated; ▼, downregulated (vs. model group induced by 80 μg/mL ox‐LDL).

* *p* < 0.05.

Figure 2C exhibited the localization analysis of the differentially expressed proteins. A considerable proportion was found in the extracellular region and nucleus, while others were associated with the plasma membrane and cytoplasm. A smaller number of proteins were localized to organelles such as the endoplasmic reticulum and peroxisomes. This distribution pattern suggests that the proteins affected by alliin treatment may participate in diverse cellular processes, particularly those related to intercellular communication, membrane dynamics, and intracellular metabolic regulation. Furthermore, as shown in Figure 2D–F, both upregulated and downregulated proteins were identified among the Alliin‐treated group, the ox‐LDL‐induced model group (80 μg/mL), and the DMEM‐treated control group, further highlighting distinct proteomic alterations associated with Alliin treatment.

### 3.3 Identification of Alliin‐Regulated Protein Networks and Pathways

**FIGURE 3:**
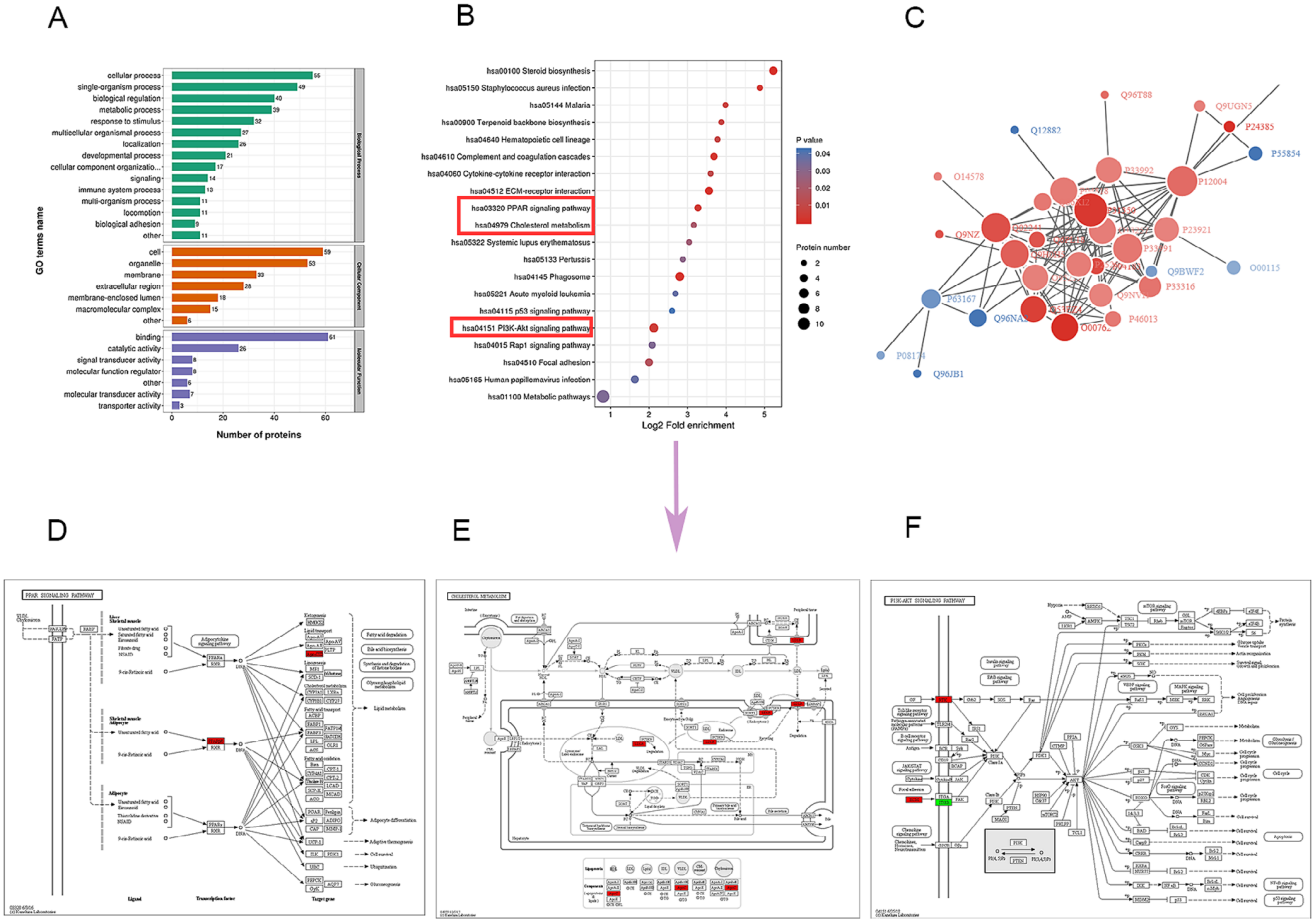
Analysis of differentially expressed proteins (A) GO functional classification enrichment distribution of differentially expressed proteins. (B) KEGG pathway enrichment bubble plot of differentially expressed proteins between the alliin‐treated group and the model group induced by 80 μg/mL ox‐LDL. (C) Protein–protein interaction network. (D) Cholesterol metabolism pathway. (E) PPAR signaling pathway. (F) PI3K/Akt signaling pathway.

Figure 3A presents the GO enrichment analysis of differentially expressed proteins in the Alliin group compared to the control group, revealing significant enrichment in several biological processes, including cellular processes, biological regulation, and metabolism. The analysis further indicated that the protective effect of alliin in HepG2 cells may involve proteins predominantly localized to membrane‐associated cellular components. In terms of molecular function, most differentially expressed proteins were classified as binding proteins.

As illustrated in Figure 3B, each bubble represents a specific pathway, with bubble size proportional to the number of proteins identified within that pathway. The color gradient, ranging from light to deep red, reflects the statistical significance of the protein expression changes, where deeper red indicates greater significance. The KEGG pathway enrichment map (Figure 3B) shows that differentially expressed proteins between the alliin‐treated and ox‐LDL‐treated groups were mainly enriched in 20 distinct pathways, three of which are associated with anti‐atherosclerotic mechanisms: the PPAR signaling pathway, cholesterol metabolism, and the PI3K/Akt signaling pathway. These three pathways are highlighted with red boxes. Figure 3D–F present visual analyses of these three significantly modulated pathways.

In Figure 3C, circles represent differentially expressed proteins, with colors indicating expression status (blue for downregulated, red for upregulated). Circle size corresponds to the number of interacting partners, with larger circles denoting proteins that have more interactions and are therefore more central within the network. To clearly display protein–protein interaction relationships, the 50 closest interacting protein pairs were selected to construct the interaction network.

As depicted in Figure 3D, red bars represent upregulated proteins, suggesting that alliin modulates cholesterol metabolism by upregulating the expression of low‐density lipoprotein receptor (LDLR). Similarly, in Figure 3E, alliin regulates the PPAR signaling pathway by upregulating the expression of PPARβ/δ. Correspondingly, in Figure 3F, alliin affects the PI3K/Akt signaling pathway by upregulating the expression of endoplasmic reticulum membrane protein complex (EMC) and receptor tyrosine kinase (RTK), while downregulating integrin beta (ITGB).

### 3.4 Western Blot Verification

**FIGURE 4:**
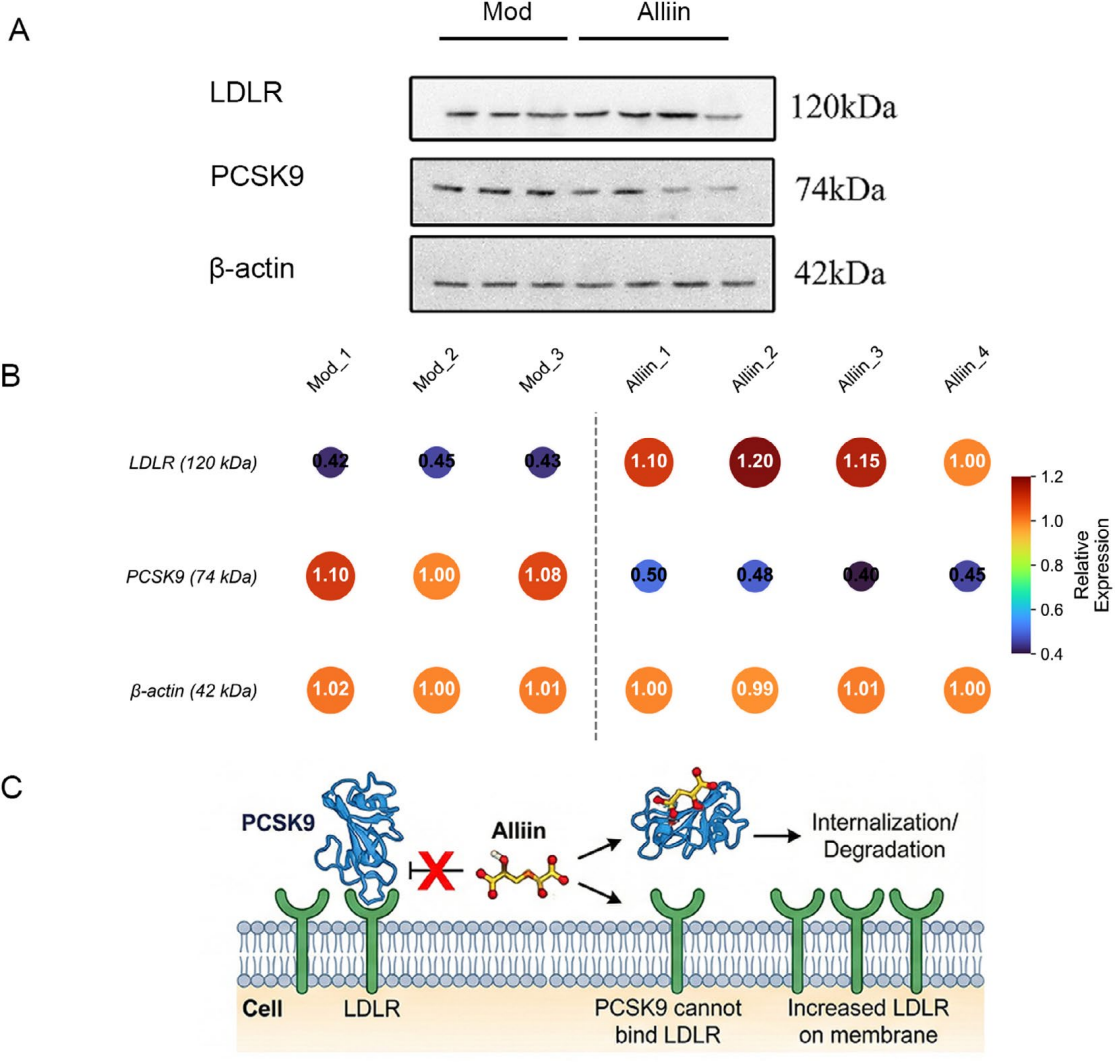
Effects of alliin on LDLR and PCSK9 protein expression and their molecular interaction (A) Western blot analysis showing that alliin treatment reduces PCSK9 expression while increasing LDLR expression (B) LDLR and PCSK9 protein levels in the control and experimental groups were analyzed by Western blotting using β‐actin as a loading control; quantified results are presented as a bubble plot (C) Schematic diagram illustrating the molecular interaction among LDLR, PCSK9, and alliin. The model group was treated with 80 μg/mL ox‐LDL, while the alliin‐treated group received 25 mg/L alliin. Data are presented as mean ± SD (*n* = 3).

As shown in Figure 4A, western blot analysis demonstrated that treatment with 25 mg/L alliin significantly increased LDLR protein expression while markedly decreasing PCSK9 expression compared with the mod group. These findings suggest that alliin may enhance LDLR levels by suppressing PCSK9 expression. Quantitative analysis of Western blot data further confirmed these observations, as illustrated by the bubble plot in Figure 4B. In this plot, redder colors indicate higher relative protein expression levels, whereas bluer colors represent lower expression levels. In addition, a schematic diagram (Figure 4C) was constructed to depict the potential molecular interaction among alliin, PCSK9, and LDLR, indicating that alliin may regulate LDLR expression through modulation of PCSK9.

## 4 Discussion

Alliin, characterized by the chemical structure S‐allyl‐L‐cysteine sulfoxide, was first identified by Stoll and Seebeck in 1947 and is recognized as the primary active component of garlic due to its lipid‐lowering, anti‐inflammatory, and antioxidant properties (Lupo et al. 2020; Mizutani et al. 2022). In uncut garlic cloves, alliin resides within the cytoplasm; however, upon cutting the cloves, it generates the pungent compound allicin through the action of alliinase (El‐Saber Batiha et al. 2020; Chen et al. 2021; Wu et al. 2020). One study demonstrated that alliin significantly mitigated increases in plasma and liver cholesterol levels in SD rats subjected to a diet containing 10% hydrogenated coconut oil, 1% cholesterol, and 0.2% cholic acid (Siegers et al. 1999). Sánchez‐Sánchez et al. (2020) reported that alliin effectively ameliorates lipid metabolism disorders in diet‐induced C57BL/6J obese mice. This study systematically investigated the lipid‐lowering and anti‐atherosclerotic effects of alliin using a proteomics‐based approach in HepG2 cells. The results demonstrate that alliin significantly reduces intracellular TC and TG levels and modulates key proteins involved in cholesterol metabolism, particularly the PCSK9/LDLR axis.

Compared to traditional experimentation, proteomics focuses on quantitative analyses of all proteins present in biological samples while elucidating biological information. In this era of systems biology and data integration, proteomic data offers novel insights into understanding the pharmacological properties of medications (Hanash 2003; Matthiesen and Carvalho 2020; Eligini et al. 2023). The KEGG pathway enrichment study revealed three signaling pathways that are strongly connected with alliin's anti‐atherosclerotic effects: the PPAR signaling pathway, cholesterol metabolism, and the PI3K/Akt signaling pathway. Wang et al. (2017) observed that alliin protects BALB/c mice from acute lung damage caused by lipopolysaccharide through increasing peroxisome proliferator‐activated receptor γ (PPARγ) expression. The activation of PPARγ is correlated with the preservation of endothelial function. As endothelial dysfunction is a key element in the development of AS, PPARγ could be a therapeutic target for AS. Moreover, ECM‐receptor interactions play an essential role in regulating cellular and organ functions. Jiang et al. concluded that ECM‐receptor interactions are involved in modulating oxidative low‐density lipoprotein‐induced endothelial cell senescence to prevent AS (Jiang et al. 2020). Similarly, the PI3K/Akt signaling pathway contributes to reducing atherosclerosis by improving endothelial cell function. Notably, several key enzymes involved in cholesterol biosynthesis (e.g., HMGCS1, SQLE, and MSMO1) were upregulated, suggesting a potential compensatory response to intracellular cholesterol reduction (Yu, Zheng, and Zhang 2021). This phenomenon has been reported in previous studies, where decreased intracellular cholesterol levels trigger feedback activation of cholesterol synthesis pathways.

AS is the consequence of a multi‐step process that eventually leads to cardiovascular disease, which is associated with high morbidity and mortality rates. Changes in lipid metabolism are risk factors for AS. Early epidemiological studies have indicated that LDLR‐mediated clearance of circulating LDL plays a critical role in reducing AS risk (Ference et al. 2017). LDL is abundant in free cholesterol, constituting 50% of its total weight and serving as the primary agent responsible for cholesterol transport (Narayan 2021; Shang et al. 2019). The LDLR, located on hepatocyte surfaces, specifically binds to LDL and subsequently internalizes LDL‐cholesterol via endocytosis, thereby reducing plasma LDL‐cholesterol levels and preventing excessive accumulation within the vascular wall that leads to AS lesions (Brandsma et al. 2019; Hartley et al. 2019).

**FIGURE 5:**
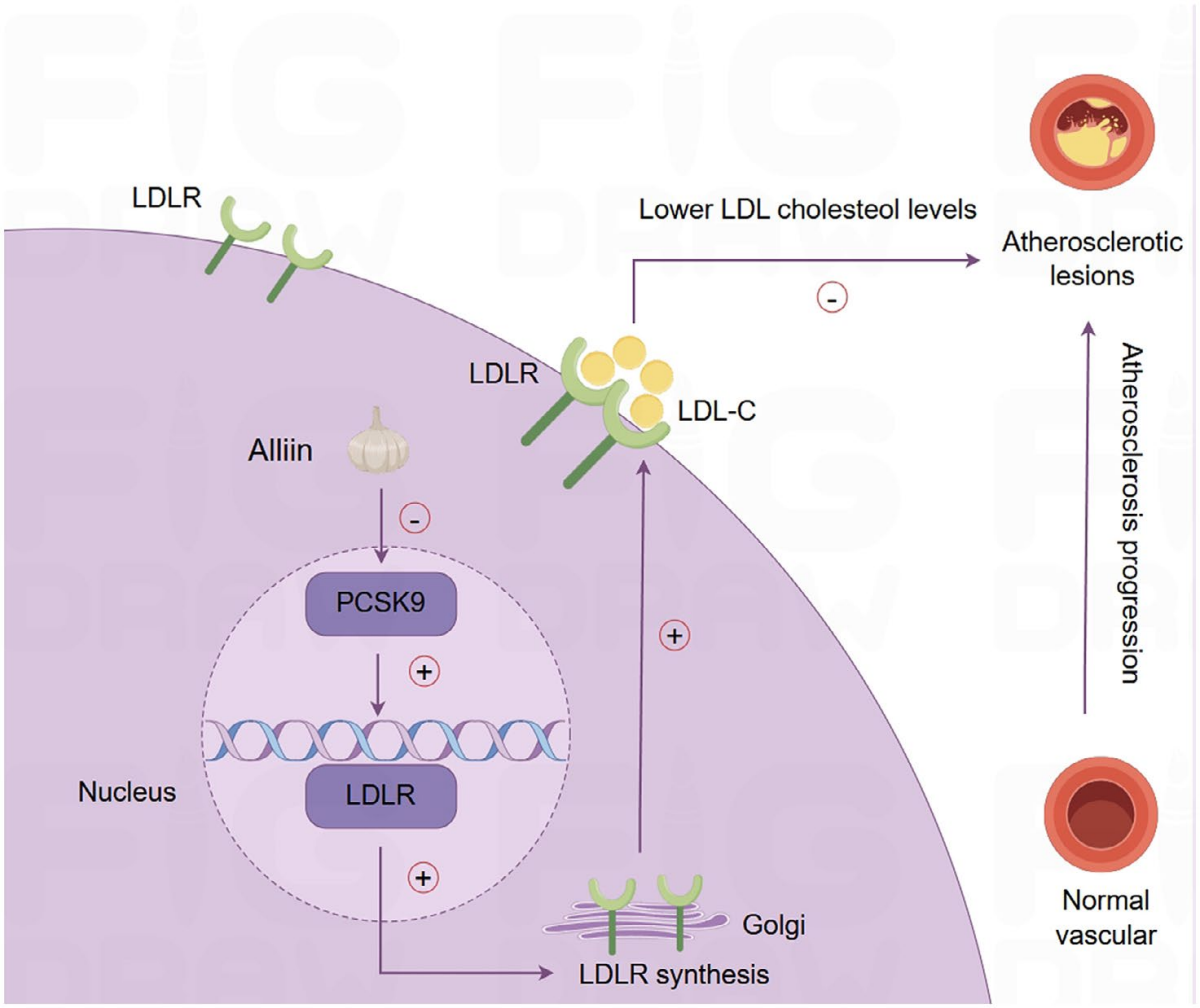
Schematic illustration of the anti‐atherosclerotic mechanism of alliin via the PCSK9/LDLR pathway.

Garlic‐derived organosulfur compounds, including S‐Ethyl‐L‐cysteine, alliin, and diallyl trisulphide, have been shown to decrease PCSK‐9 activity, increase LDLR function, and lower LDL cholesterol levels (Rouf et al. 2020; Chen et al. 2004). In our research, both proteomic data and Western blot validation consistently showed that alliin upregulated LDLR expression while downregulating PCSK9 (Figure 5). PCSK9 is a critical regulator of LDLR degradation, and its inhibition leads to increased LDLR availability on the hepatocyte surface, thereby enhancing LDL clearance. Therefore, modulation of the PCSK9/LDLR axis represents a well‐established therapeutic strategy for lowering cholesterol levels. Our findings suggest that alliin may exert its lipid‐lowering effect through this pathway.

Currently, Statins remain the primary pharmacological agent for clinical management of AS (Sadowska et al. 2023). While HMG‐CoA reductase inhibitors are known to lower lipid levels, they also impede the normal physiological functions of this enzyme, which may pose long‐term health risks. This investigation holds significant social implications by exploring the pharmacodynamic basis and mechanisms underlying alliin's anti‐AS effects.

However, several limitations should be acknowledged. First, this study was conducted exclusively in HepG2 cells, which are derived from hepatocellular carcinoma and may not fully reflect normal hepatocyte physiology. Second, although proteomics provides a global view of protein changes, only the PCSK9/LDLR pathway was experimentally validated, and other pathways require further confirmation. Third, the in vitro nature of this study limits the direct translation of these findings to in vivo conditions.

Previous studies have demonstrated that ox‐LDL is primarily taken up by scavenger receptors, such as CD36 and scavenger receptor class A, rather than by LDLR (Levitan et al. 2010; Moore and Freeman 2006). In the present study, although it demonstrates that alliin alleviates lipid accumulation and regulates cholesterol metabolism in HepG2 cells, potentially through modulation of the PCSK9/LDLR pathway. However, these effects are more likely related to intracellular cholesterol homeostasis rather than direct involvement in ox‐LDL uptake.

## 5 Conclusions

Our research shows that alliin was shown to reduce intracellular lipid accumulation and alter the proteomic profile in ox‐LDL‐induced HepG2 cells. Proteomic and bioinformatics analyses indicated that the observed changes are associated with pathways related to cholesterol metabolism, PPAR, and PI3K/Akt signaling pathways. Furthermore, experimental validation demonstrated that alliin regulates the PCSK9/LDLR axis, suggesting a role in modulating intracellular cholesterol homeostasis. It should be noted that the PCSK9/LDLR pathway is based on proteomic associations and has not been mechanistically validated with any type of interventional experiment. Further studies, particularly in vivo investigations, are required to determine whether these effects can be translated to systemic lipid regulation or atherosclerosis‐related outcomes.

## Author Contributions

**Yuanyuan Tang:** conceptualization, investigation, funding acquisition, writing – original draft, validation, methodology, software, formal analysis. **Xiaoshi Liu:** conceptualization, investigation, validation, methodology, project administration, formal analysis, software.

## Funding

This work was supported by the 2025 Chengdu Municipal–University–Hospital Collaborative Innovation Fund Project (No. WXLH202501111 to Yuanyuan Tang) and the Sichuan Cancer Hospital Excellent Youth Fund (No. YB2024013 to Xiaoshi Liu).

## Ethics Statement

The authors have nothing to report.

## Conflicts of Interest

The authors declare no conflicts of interest.

## Supporting information


**Table S1:** MS‐based identification information of differentially expressed proteins.

## Data Availability

The data that support the findings of this study are available from the corresponding author upon reasonable request.
